# Dr Elisha Baker

**Published:** 1872-05

**Authors:** J. G. Ambler, Wm. H. Atkinson, J. H. Foster, Charles Merritt

**Affiliations:** Committee.; Committee.; Committee.; Committee.


### OBITUARY.

*Dr. Elisha Baker.*—At a meeting of the Dental Profession, convened on Saturday afternoon, March 9th, 1872, at the residence of Dr. J. G. Ambler, 25 West 23rd street, New York, relative to the death of ELISHA BAKER, M. D., (which took place the day previous), Dr. F. H. Clark was called to the chair, and Dr. Charles Merritt, elected Secretary.

Dr. Ambler explained the objects of the call, viz: to take some action as a body regarding the death of Dr. Baker. After the very handsome tribute paid to his memory by those present, it was on motion—

*Resolved*, That our heartfelt sympathies are tendered the family of the deceased, and that we will manifest our affection and appreciation of his many virtues, by attending his funeral. It was further—

*Resolved*, That Drs. J. G. Ambler, Wm. H. Atkinson, J. H. Foster and Charles Merritt, be a committee to draft suitable resolutions, expressive of the sense of the profession, present a copy to the family of deceased, and publish in the dental journals,

CHARLES MERRITT, Secretary.

WHEREAS, Death is at no time a welcome visitor, and most unwelcome when a bright and shining light, an honored and revered citizen is its victim.

*We* are this day called to mourn the loss of one highly esteemed in the community in which he lived, a pioneer in the profession of Dentistry, one who by precept and example labored to elevate the standard of professional excellence, during an unprecedented series of over forty years, and his retirement from active practice some fifteen years since, carried with him the universal respect, esteem and regard of his professional brethren. Therefore

*Resolved*, That while bowing in humble submission to the decree of Him, who doeth all things well, and with hearts overflowing with gratitude, that He did vouchsafe to us such a man and that he has been permitted to bear such a rich legacy. We would place in record, these expressions of our bereavement and sorrow, with the assurance that the name of Elisha Baker, will long stand on the historic page, as one of the brightest ornaments of our profession.

*Resolved*, That Dr. J. G. Ambler be requested to prepare and deliver a eulogy on the deceased, and for publication.

(Signed.)

J. G. AMBLER,

WM. H. ATKINSON,

J. H. FOSTER,

CHARLES MERRITT.

} *Committee.*

